# Corrected Thermodynamics of Black Holes in *f*(*R*) Gravity with Electrodynamic Field and Cosmological Constant

**DOI:** 10.3390/e26100868

**Published:** 2024-10-15

**Authors:** Mou Xu, Yuying Zhang, Liu Yang, Shining Yang, Jianbo Lu

**Affiliations:** Department of Physics, Liaoning Normal University, Dalian 116029, China

**Keywords:** *f*(*R*) theory, corrected entropy, corrected black hole thermodynamics, stability

## Abstract

The thermodynamics of black holes (BHs) and their corrections have become a hot topic in the study of gravitational physics, with significant progress made in recent decades. In this paper, we study the thermodynamics and corrections of spherically symmetric BHs in models $f\left(R\right)=R+\alpha R^{2}$ and $f\left(R\right)=R+2\gamma \sqrt{R+8\Lambda }$ under the f(R) theory, which includes the electrodynamic field and the cosmological constant. Considering thermal fluctuations around equilibrium states, we find that, for both f(R) models, the corrected entropy is meaningful in the case of a negative cosmological constant (anti-de Sitter–RN spacetime) with Λ=−1. It is shown that when the BHs’ horizon radius is small, thermal fluctuations have a more significant effect on the corrected entropy. Using the corrected entropy, we derive expressions for the relevant corrected thermodynamic quantities (such as Helmholtz free energy, internal energy, Gibbs free energy, and specific heat) and calculate the effects of the correction terms. The results indicate that the corrections to Helmholtz free energy and Gibbs free energy, caused by thermal fluctuations, are remarkable for small BHs. In addition, we explore the stability of BHs using specific heat. The study reveals that the corrected BH thermodynamics exhibit locally stable for both models, and corrected systems undergo a Hawking–Page phase transition. Considering the requirement on the non-negative volume of BHs, we also investigate the constraint on the EH radius of BHs.

## 1. Introduction

The essence of general relativity (GR) is a theory of gravity in the framework of relativity. It is an inevitable result of the development of Newton’s gravity theory and special relativity (SR). In 1687, Newton proposed the first complete theory of gravity, the law of universal gravitation, based on the research of Kepler, Galileo, and others. This is a successful theory that applied to the motion of low-speed objects in a weak gravitational field. In subsequent research, it was found that Newton’s theory of gravity has some problems [1,2], e.g., the equivalent problem between the gravitational mass and the inertial mass, the Neumann–Zeiliger paradox, and the theoretical explanation for the precession of Mercury’s perihelion. In addition, in special relativity, the covariance of physical laws needs to be limited in the inertial reference frame, i.e., the principle of relativity, reflecting the special status of the inertial reference frame. To address the aforementioned issues, in 1915, Einstein established the theory of general relativity based on the equivalence principle and the general covariance principle. Since its proposal, GR has received widespread attention and has been supported by many experimental observations, such as the precession of Mercury’s perihelion [3,4], gravitational lensing [5], test for equivalence principle [6], gravitational deflection of light [7], direct images of the BH shadows from the Event Horizon Telescope group [8,9]. Especially in recent years, gravitational waves, predicted by GR, were also confirmed by the LIGO-Virgo collaborative experiment [10,11].

Furthermore, GR also faces some challenges, e.g., issues on the inflation in the early universe [12], accelerated expansion or dark energy in the late universe [13,14], dark matter problem [15], and quantization of gravity [16]. Therefore, there are some alternative and extended theories on GR, such as the f(R) theory (*R* is the Ricci scalar) [16,17,18], f(R,T) theory, (where *T* is the trace of the energy-momentum tensor) [19,20,21,22], f(G) theory (*G* is the Gauss–Bonnet invariant) [23,24]. In particular, the f(R)-modified gravity theory has received much attention from researchers in recent decades. Within the framework of this theory, scholars have explored issues such as early universe inflation [25,26], gravitational wave physics [27,28], the stability of BHs [29,30], and others.

Regarding the study of f(R) gravity, a large number of theoretical models have been established [31,32,33,34]. Here, we focus on two representative models. One is the first feasible cosmological inflation model $f\left(R\right)=R+\alpha R^{2}$, established by Starobinsky in 1980, which explains the early inflation of the universe from a phenomenological perspective [26]. Extensive research has been conducted within the framework of this theoretical model, such as the properties of the universe in the Palatini formalism [35,36], black bounce solution [37] and the astronomical observation constraints [38]. Another model is denoted as $f\left(R\right)=R+2\gamma \sqrt{R+8\Lambda }$ (Λ is the cosmological constant) [39]. Reference [39] explored the thermodynamic properties of BHs under these two f(R) models and the equivalence of thermodynamic quantities in Jordan and Einstein frames.

Since the pioneering work of Bekenstein and Hawking, the study of the thermodynamics of BHs has flourished as an important area of research in modern physics. The thermodynamic properties of BHs are usually manifested through the behavior of entropy, temperature, and other thermodynamic variables. The thermodynamics of different types of BHs have been considered and extensively studied by researchers from different perspectives, such as three-dimensional charged BHs [40,41], anti-de Sitter BHs [42,43], spherically symmetric charged BHs [44], and others [45,46,47,48,49,50,51,52].

According to quantum mechanics, Hawking radiation (HR) can occur for BHs [53,54]. Because of HR, BHs shrink in size, which may lead to evaporation [55,56]. Further, the evaporation of BHs may leave observational signatures [57,58], and the study on the experimental observations of HR are available in the literature [59]. Considering quantum effects, a good way to investigate BHs is to explore their thermal fluctuations [60,61,62,63], which can help us understand the microscopic origin of entropy [64]. In general, entropy correction is important when the size of a BH is reduced by HR and its temperature is increased [56], which is useful for solving questions on the quantum fluctuations associated with the study of BH thermodynamics [65]. The logarithmic correction is a widely accepted correction form of BH entropy [66,67,68,69,70,71], which could be interpreted as a quantum effect [72,73], coming from thermal fluctuations and yielding to the modification of the holographic principle [74,75]. In particular, the logarithmic correction of entropy is indeed important when the BH is small; for large BHs, the correction can be ignored, as the thermal fluctuations may not occur in it [65]. As a perturbation correction, it is thought that it can be used to test quantum gravity [76,77,78,79,80], so it makes sense to explore the correction entropy of BHs under different theoretical models. In recent years, more and more attention has been paid to the study of corrected BH thermodynamics. The study of thermodynamics in the presence of correction terms can provide important information about the relevant properties of BHs. Corrections to BH thermodynamics have been widely explored, including rotating and charged BTZ BHs [81], massive BHs in AdS space [82,83], Godel BHs [84], and expanding BHs [85].

In this paper, we consider relevant issues to corrected thermodynamics of static spherically symmetric BHs under the f(R) gravity theory, in the presence of small thermal fluctuations around the equilibrium point. This paper is organized as follows. In Section 2, in the framework of the f(R)-modified theory containing the electrodynamic field and the cosmological constant, we briefly summarize two representative gravitational models and present the fundamental thermodynamic quantities of their static spherically symmetric BH solutions. The corrected entropy and corrected thermodynamics of BHs under both f(R) models are studied in Section 3. Section 4 outlines the conclusion. In this paper, the Planck units (fundamental constants) $\hbar =k_{B}=G=c=1$ are used.

## 2. Thermodynamics of Spherically Symmetric BHs in f(R) Gravity Theory with Electrodynamic Field and Cosmological Constant

In this section, we begin with a brief description of the basic equations in the framework of the f(R) gravity theory containing the cosmological constant and the electrodynamic field. In absence of ordinary matter, the action of a system is written as [16,39,44,86,87]:

(1) $$\begin{array}{c} S_{t}=S_{g}+S_{e.m.}. \end{array}$$

Here, $S_{g}$ is the four-dimensional gravitational action in the f(R) theory:

(2) $$\begin{array}{c} S_{g}=\frac{1}{2\kappa }\int d^{4}x\sqrt{-g}\left[f\left(R\right)-\Lambda \right], \end{array}$$

where Λ is the cosmological constant [86,87], and it realizes the accelerated expansion of universe, which is required by the cosmic observations. *R* is the Ricci scalar, κ=8πG (*G* is Newton’s gravitational constant), *g* is the determinant of the metric tensor $g_{\mu \nu }$, and f(R) is an arbitrarily differentiable function of *R*. The electrodynamic action $S_{e.m.}$ is [39,44]:

(3) $$\begin{array}{c} S_{e.m.}=-\frac{1}{2}F^{2}=-\frac{1}{2}F_{\mu \nu }F^{\mu \nu }, \end{array}$$

where $F_{\mu \nu }=2A_{\left[\mu ,\nu \right]}$ is the anti-symmetric electromagnetic tensor and $A_{\mu }$ is the 1-form gauge potential [88,89]. The square bracket denotes anti-symmetrization, i.e., $A_{\left[\mu ,\nu \right]}=\frac{1}{2}\left(A_{\mu ,\nu }-A_{\nu ,\mu }\right)$, and the comma stands for the ordinary differentiation. The variation of action (1) relative to $g_{\mu \nu }$ and the electromagnetic field strength *F*, respectively, provide the following [39,44,90]:

(4) $$\begin{array}{c} R_{\mu \nu }f_{R}-\frac{1}{2}g_{\mu \nu }f\left(R\right)-2g_{\mu \nu }\Lambda +g_{\mu \nu }□f_{R}-\nabla_{\mu }\nabla_{\nu }f_{R}-\kappa T_{\mu \nu }=0, \end{array}$$

(5) $$\begin{array}{c} \partial_{\nu }\left(\sqrt{-g}F^{\mu \nu }\right)=0, \end{array}$$

where $f_{R}=\frac{\partial f(R)}{\partial R}$. $T_{\mu \nu }$ defines the traceless energy–momentum tensor of the electrodynamic field, as follows:

(6) $$\begin{array}{c} T_{\mu \nu }=\frac{1}{\kappa }\left(2g_{\rho \sigma }F_{\nu }^{\rho }F_{\mu }^{\sigma }-\frac{1}{2}g_{\mu \nu }F^{2}\right). \end{array}$$

The trace of Equation (4) is

(7) $$\begin{array}{c} Rf_{R}-2f\left(R\right)-8\Lambda +3□f_{R}=0. \end{array}$$

Next, we study the relevant thermodynamic quantities in their corresponding BH systems under two specific f(R) theoretical models.

### 2.1. $\mathrm{Model}\;I:\;f\left(R\right)=R+\alpha R^{2}$

The spacetime line element of the static spherical symmetry can be written as

(8) $$\begin{array}{c} ds^{2}=-N_{1}\left(r\right)dt^{2}+\frac{dr^{2}}{N_{1}\left(r\right)}+r^{2}\left(d\theta^{2}+\sin^{2}\theta d\phi^{2}\right). \end{array}$$

One should notice that, in many solutions to gravitational field equations, the form of the metric is highly constrained by symmetry and the energy–momentum content of spacetime [1]. As stated in [44], the reason to consider the form of metric (8) is to be able to find an exact solution for the model in the framework of the f(R) theory, since other forms of metric make the field equations very complicated and not easy to solve. Consider the f(R) gravity model as [26]

(9) $$\begin{array}{c} f\left(R\right)=R+\alpha R^{2} \end{array}$$

with model parameter α. Equations (4)–(8) can be solved to provide the following [39,91]:

(10) $$\begin{array}{c} N_{1}\left(r\right)=1-\frac{2M_{1}}{r}+\frac{2\Lambda r^{2}}{3}+\frac{q^{2}}{\Omega^{2}r^{2}}, \end{array}$$

where $M_{1}$ is the mass of BH, *q* is the charge, and $\Omega =\sqrt{1-16\alpha \Lambda }$. This solution corresponds to a constant Ricci scalar: R=−8Λ. For Λ>0 (de Sitter–Reissner–Nordstr$\ddot{o}$m spacetime), to ensure that the theoretical model (9) satisfies the stability and the ghost-free constraints, the range of α is limited to $0<\alpha <\frac{1}{16\Lambda }$; for Λ<0 (anti-de Sitter–Reissner–Nordstr$\ddot{o}$m spacetime), we have α<0 [39].

With this model (9), applying $N_{1}\left(r_{+}\right)=0$ yields the BH event horizon (EH) radius as a function of the total mass contained within the EH (or called the geometrical mass), as follows:

(11) $$\begin{array}{c} m_{1}=\frac{\left(3+2\Lambda r_{+}^{2}\right)\Omega^{2}r_{+}^{2}+3q^{2}}{6\Omega^{2}r_{+}}. \end{array}$$

Next, we introduce the relevant thermodynamic quantities in this model, such as the Bekenstein–Hawking entropy and the Hawking temperature. In f(R) gravity, the Bekenstein–Hawking entropy is defined as [92]

(12) $$\begin{array}{c} S=\frac{1}{4}f_{R}A, \end{array}$$

where *A* is the area of the event horizon of a BH. For obtaining a solution (10), the entropy of a BH is

(13) $$\begin{array}{c} S_{1}=\left(1-16\alpha \Lambda \right)\pi r_{+}^{2}. \end{array}$$

It can be seen that when α=0, the above equation degenerates to the corresponding form in GR. At the EH, the Hawking temperature of a BH $T_{H}$ is expressed as [93]

(14) $$\begin{array}{c} T_{H}=\frac{1}{4\pi }N_{1}^{\prime }\left(r_{+}\right), \end{array}$$

where "′" denotes the derivative with respect to the radial coordinate. Substituting the solution (10) into Equation (14), the Hawking temperature of the BH for this f(R) model is obtained [39],

(15) $$\begin{array}{c} T_{H1}=\frac{\left(1+2\Lambda r_{+}^{2}\right)\Omega^{2}r_{+}^{2}-q^{2}}{4\pi \Omega^{2}r_{+}^{3}}. \end{array}$$

The specific heat of a BH at the EH is one of the physical quantities that responds to its thermodynamic stability. Using the entropy (13) and the Hawking temperature (15), the specific heat can be given as

(16) $$\begin{array}{c} C_{1}=T_{H1}\frac{dS_{1}}{dT_{H1}}=\frac{2\pi r_{+}^{2}\Omega^{2}\left(q^{2}+r_{+}^{2}\left(1+2r_{+}^{2}\Lambda \right)\Omega^{2}\right)}{3q^{2}+r_{+}^{2}\left(-1+2r_{+}^{2}\Lambda \right)\Omega^{2}}. \end{array}$$

As a state function, free energy describes the possible amount of energy present for doing work [94]. Based on the entropy (13) and Hawking temperature (15), the specific form of the Helmholtz free energy can be found as [81]

(17) $$\begin{array}{c} F_{1}=-\int S_{1}dT_{H1}=\frac{3q^{2}}{4r_{+}}-\frac{1}{12}r_{+}\left(-3+2\Lambda r_{+}^{2}\right)\Omega^{2}. \end{array}$$

In addition, enthalpy energy can also be written in terms of entropy and the Hawking temperature:

(18) $$\begin{array}{c} H_{1}=\int T_{H1}dS_{1}=\frac{q^{2}}{2r_{+}}+\frac{1}{6}r_{+}\left(3+2\Lambda r_{+}^{2}\right)\Omega^{2}. \end{array}$$

In the study of BH thermodynamics, the variation of the cosmological constant Λ was considered [95]. Reference [96] provides the physical interpretation of Λ as pressure and defines the pressure as

(19) $$\begin{array}{c} P=-\frac{\Lambda }{8\pi }. \end{array}$$

The enthalpy (18) can be rewritten according to the pressure (19) as follows:

(20) $$\begin{array}{c} H_{1}=\frac{q^{2}}{2r_{+}}+\frac{1}{6}r_{+}\left(3-16P\pi r_{+}^{2}\right)\left(1+128P\pi \alpha \right). \end{array}$$

The conjugate quantity to the pressure is the volume [97]; the volume of this thermodynamic system can be calculated as

(21) $$\begin{array}{c} V_{1}=\frac{dH_{1}}{dP}=64\pi r_{+}\alpha -\frac{8}{3}\pi r_{+}^{3}\left(1+256P\pi \alpha \right). \end{array}$$

From the thermodynamic quantities derived above, we can calculate the internal energy and Gibbs free energy of the BH. According to Equations (18)–(21), we can obtain the expression for internal energy as follows:

(22) $$\begin{array}{c} U_{1}=H_{1}-PV_{1}=\frac{q^{2}}{2r_{+}}-64P\pi r\alpha +\frac{8}{3}P\pi r_{+}^{3}\left(1+256P\pi \alpha \right)-\frac{1}{6}r\left(3+2r_{+}^{2}\Lambda \right)\left(-1+16\alpha \Lambda \right). \end{array}$$

In thermodynamics, Gibbs free energy measures the maximum value of mechanical work [94]. With the help of the Helmholtz free energy (17), pressure (19) and volume (21) given above, we can carry out a calculation to obtain Gibbs free energy as follows:

(23) $$\begin{array}{c} G_{1}=F_{1}+PV_{1}=\frac{3q^{2}}{4r_{+}}+\frac{r_{+}}{4}-4r_{+}\alpha \Lambda -\frac{1}{6}r_{+}{‌}^{3}\left(16P\pi +\Lambda \right)\left(1+256P\pi \alpha -16\alpha \Lambda \right). \end{array}$$

### 2.2. $\mathrm{Model}\;\mathrm{II}:\;f\left(R\right)=R+2\gamma \sqrt{R+8\Lambda }$

In this subsection, we consider the static spherically symmetric metric and the f(R) model, respectively, as follows [39]:

(24) $$\begin{array}{c} ds^{2}=-N_{2}\left(r\right)dt^{2}+\frac{dr^{2}}{N_{2}\left(r\right)}+r^{2}\left(d\theta^{2}+\sin^{2}\theta d\phi^{2}\right), \end{array}$$

(25) $$\begin{array}{c} f\left(R\right)=R+2\gamma \sqrt{R+8\Lambda }, \end{array}$$

where γ is a nonzero constant in the model, and Λ is the cosmological constant [39,44]. Under the line element (24), reference [39] was used to calculate and provide the solution in the following form:

(26) $$\begin{array}{c} N_{2}\left(r\right)=\frac{1}{2}-\frac{2M_{2}}{r}+\frac{2\Lambda r^{2}}{3}+\frac{q^{2}}{r^{2}}, \end{array}$$

where the mass of a BH $M_{2}=-\frac{c_{1}}{6\gamma }$ with a constant parameter $c_{1}$. To be consistent with the description in [39], one can take $c_{1}=1$ for simplicity. Thus, the parameter γ will be restricted to negative value. Following reference [38], in contrast to the earlier study [39], the charge *q* associated with the current solution does not depend on the model parameter γ. Based on the stability conditions, the range of the parameter γ is given as follows [39]: γ<0. Since the parameter γ≠0, the solution cannot be returned to the corresponding form in GR. Additionally, in this spacetime geometry, the Ricci scalar is not constant: $R=-8\Lambda +\frac{1}{r^{2}}$. The application of this model in cosmology can be found in reference [98]. For the relevant studies on BHs in this kind of f(R) model, one can see [99,100,101], where exact black hole solutions were provided along with methods to probe the physics of the non-linearity of f(R).

In this model, we also calculate the horizon mass, Bekenstein–Hawking entropy, Hawking temperature, specific heat, Helmholtz free energy, enthalpy, volume, internal energy, and Gibbs free energy of the horizon, as follows:

(27) $$\begin{array}{c} m_{2}=\frac{6q^{2}+3r_{+}^{2}+4\Lambda r_{+}^{4}}{12r_{+}}, \end{array}$$

(28) $$\begin{array}{c} S_{2}=\pi r_{+}^{2}\left(1+\gamma r_{+}\right), \end{array}$$

(29) $$\begin{array}{c} T_{H2}=\frac{r_{+}^{2}+4\Lambda r_{+}^{4}-2q^{2}}{8\pi r_{+}^{3}}, \end{array}$$

(30) $$\begin{array}{c} C_{2}=\frac{\pi r_{+}^{2}\left(2+3r_{+}\gamma \right)\left(-2q^{2}+r_{+}^{2}+4r_{+}^{4}\Lambda \right)}{6q^{2}-r_{+}^{2}+4r_{+}^{4}\Lambda }, \end{array}$$

(31) $$\begin{array}{c} F_{2}=\frac{1}{8}\left(\frac{6q^{2}}{r_{+}}+r_{+}+\frac{r_{+}^{2}\gamma }{2}-\frac{4r_{+}^{3}\Lambda }{3}-r_{+}^{4}\gamma \Lambda -6q^{2}\gamma \log\left(r_{+}\right)\right), \end{array}$$

(32) $$\begin{array}{c} H_{2}=\frac{1}{8}\left(\frac{4q^{2}}{r_{+}}+2r_{+}+\frac{3r_{+}^{2}\gamma }{2}+\frac{8r_{+}^{3}\Lambda }{3}+3r_{+}^{4}\gamma \Lambda -6q^{2}\gamma \log\left(r_{+}\right)\right), \end{array}$$

(33) $$\begin{array}{c} V_{2}=\frac{1}{8}\left(-\frac{64\pi r_{+}^{3}}{3}-24\pi r_{+}^{4}\gamma \right), \end{array}$$

(34) $$\begin{array}{c} U_{2}=\frac{1}{16}\left(\frac{8q^{2}}{r_{+}}+r_{+}\left(4+3r_{+}\gamma \right)-12q^{2}\gamma \log\left(r_{+}\right)\right), \end{array}$$

(35) $$\begin{array}{c} G_{2}=\frac{1}{48}r_{+}\left(6+3r_{+}\gamma -18q^{2}\left(2+r_{+}\gamma \right)+8r_{+}^{2}\Lambda +12r_{+}^{3}\gamma \Lambda \right). \end{array}$$

## 3. Corrected Thermodynamics of Spherically Symmetric BHs in the Framework of f(R)-Modified Theory

### 3.1. Corrected Entropy of Black Holes

In this section, we explore entropy correction caused by small thermal fluctuations around the equilibrium state. To calculate the corrected entropy, the partition function of the canonical ensemble is usually defined as

(36) $$\begin{array}{c} Z\left(\beta \right)=\int_{0}^{\infty }\rho \left(E\right)e^{-\beta E}dE, \end{array}$$

where $\beta =\frac{1}{T_{H}}$ for a given partition function. The density of states ρ(E) can be calculated through the inverse Laplace transform of (36):

(37) $$\begin{array}{c} \rho \left(E\right)=\frac{1}{2\pi i}\int_{\beta_{0}-i\infty }^{\beta_{0}+i\infty }e^{S(\beta )}d\beta , \end{array}$$

where

(38) $$\begin{array}{c} S(\beta )=\ln Z(\beta )+\beta E, \end{array}$$

is the exact entropy of a BH. This exact entropy, associated with temperature, is the sum of the entropies of all individual subsystems. To study the effect of thermal fluctuations on entropy, we perform a Taylor expansion of S(β) around the equilibrium point $\beta =\beta_{0}$:

(39) $$\begin{array}{c} S\left(\beta \right)=S_{0}+{\left.\frac{1}{2}{\left(\beta -\beta_{0}\right)}^{2}\frac{\partial^{2}S}{\partial \beta^{2}}\right|}_{\beta =\beta_{0}}+\ldots , \end{array}$$

where $S_{0}$ is the Bekenstein–Hawking entropy. Substituting Equation (39) into Equation (37), we get

(40) $$\begin{array}{c} \rho \left(E\right)=\frac{e^{S_{0}}}{2\pi i}\int_{\beta_{0}-i\infty }^{\beta_{0}+i\infty }e^{\left[{\left.\frac{1}{2}{\left(\beta -\beta_{0}\right)}^{2}\frac{\partial^{2}S}{\partial \beta^{2}}\right|}_{\beta =\beta_{0}}\right]}d\beta . \end{array}$$

A further derivation of Equation (40) yields

(41) $$\begin{array}{c} \rho \left(E\right)=\frac{e^{S_{0}}}{\sqrt{{\left.2\pi \frac{\partial^{2}S}{\partial \beta^{2}}\right|}_{\beta =\beta_{0}}}}, \end{array}$$

and the corrected entropy caused by thermal fluctuations is expressed as

(42) $$\begin{array}{c} S\left(\beta \right)=\ln\rho \left(E\right)=S_{0}-{\left.\frac{1}{2}\ln\frac{\partial^{2}S}{\partial \beta^{2}}\right|}_{\beta =\beta_{0}}+\ldots . \end{array}$$

Here, we consider neglecting higher-order correction terms. Equation (42) can play a significant role for thermodynamic systems (e.g., BH systems) [94]. To further simplify the above formula, following the method shown in refs. [94,102,103,104,105], with which we can obtain

(43) $$\begin{array}{c} {\left.\frac{\partial^{2}S}{\partial \beta^{2}}\right|}_{\beta =\beta_{0}}=S_{0}T_{H}^{2} \end{array}$$

with a parameterized form of the entropy function [102,103]:

(44) $$\begin{array}{c} S\left(\beta \right)=a\beta^{m}+b\beta^{-n}, \end{array}$$

where all the constants satisfy m,n,a,b>0, and m=1, n=1 can be taken as a special example. Thus, the corrected entropy of a BH in this case is expressed as

(45) $$\begin{array}{c} S_{c}=S_{0}-\frac{1}{2}\ln S_{0}T_{H}{‌}^{2}. \end{array}$$

For the two classes of f(R) models considered in this paper, $f\left(R\right)=R+\alpha R^{2}$ and $f\left(R\right)=R+2\gamma \sqrt{R+8\Lambda }$, the specific forms of the corresponding BH corrected entropies are written, respectively, as follows:

(46) $$\begin{array}{c} S_{c1}=\Omega^{2}\pi r_{+}^{2}-\frac{1}{2}\ln\left(\frac{\Omega^{2}{\left(q^{2}-r_{+}^{2}\left(1+2r_{+}^{2}\Lambda \right)\Omega^{2}\right)}^{2}}{16\pi \Omega^{4}r_{+}^{4}}\right), \end{array}$$

(47) $$\begin{array}{c} S_{c2}=\left(1+r_{+}\gamma \right)\pi r_{+}{‌}^{2}-\frac{1}{2}\ln\left(\frac{\left(1+r_{+}\gamma \right){\left(-2q^{2}+r_{+}^{2}+4r_{+}^{4}\Lambda \right)}^{2}}{64\pi r_{+}^{4}}\right). \end{array}$$

**Figure 1:**
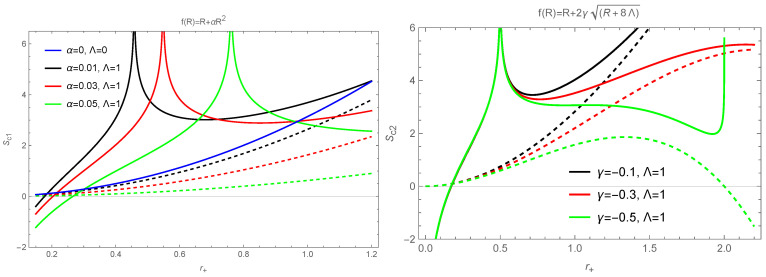

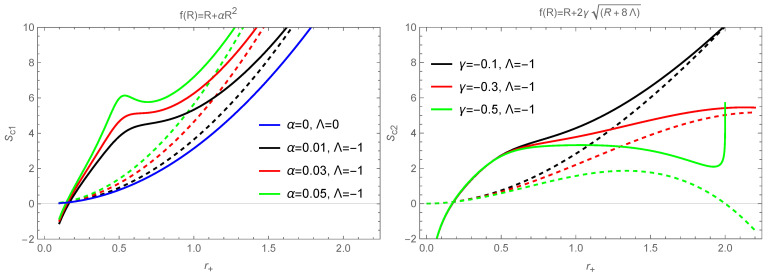
Variation of entropy with the horizon radius for two classes of spherically symmetric BH solutions in the f(R) theory. The dashed line represents the Bekenstein–Hawking entropy, and the solid line represents the corrected entropy. In the upper pictures, Λ=1; in the lower pictures, Λ=−1. For both models, we set q=0.5. The blue curve in the left panel represents the result for the RN BH in GR.

Based on Equations (13), (28), (46) and (47), we plot entropy as a function of the horizon radius for the two f(R) models in Figure 1, where Λ>0,Λ<0 are considered to plot pictures, respectively. As an example, the values of model parameters are taken as Λ=1 (or −1) and q=0.5. As can be seen from Figure 1 (upper), for the case of Λ=1, in order to ensure that the logarithmic function is meaningful, it is clearly necessary to require that the $\frac{\Omega^{2}{\left(q^{2}-r_{+}^{2}\left(1+2r_{+}^{2}\Lambda \right)\Omega^{2}\right)}^{2}}{16\pi \Omega^{4}r_{+}^{4}}>0$ for Model I and $\frac{\left(1+r_{+}\gamma \right){\left(-2q^{2}+r_{+}^{2}+4r_{+}^{4}\Lambda \right)}^{2}}{64\pi r_{+}^{4}}>0$ for Model II, which result in the localized divergence of the corrected entropy. In addition, consider that the corrected entropy is given based on a series expansion and associated with small thermal fluctuations, which leads to the corrected entropy only applying to a specific $r_{+}$ range. This further puts a tight restriction on $r_{+}$ and BHs. For the case Λ=−1, we see from the figure that the corrected entropy reflects a correction that is small relative to the Bekenstein–Hawking entropy and does not diverge. It seems that for both f(R) models, the corrected entropy is meaningful for case of negative cosmological constant (anti-de Sitter–RN spacetime) with Λ=−1. Moreover, it is shown that when the BHs’ horizon radius is small, thermal fluctuations have a more significant effect on the corrected entropy. Also, from Figure 1 (lower), we can observe that compared to the Bekenstein–Hawking entropy, the corrected entropy for two models with parameter Λ=−1 contains richer physical information, where, at the location of the small $r_{+}$, the negative corrected entropy can be emerged, which corresponds to a repulsive effect of gravity via the negative effective Newtonian gravitational constant.

### 3.2. Corrected Thermodynamics of Black Holes

In this section, we explore the corrected thermodynamics of BHs under the two classes of f(R) models considered above. We investigate the effect of small thermal fluctuations on the correction of thermodynamic quantities such as internal energy, entropy, enthalpy, Helmholtz free energy, pressure, specific heat, and Gibbs free energy by comparing the behavior of the original and corrected thermodynamic quantities. As shown in the figure above, considering that it is more meaningful for the corrected entropy of the two f(R) models in the case of Λ=−1, we plot the pictures of the corrected thermodynamic quantities by choosing Λ=−1 for discussion in the following section.

#### 3.2.1. $\mathrm{Model}\;I:\;f\left(R\right)=R+\alpha R^{2}$

First, we calculate the corrected enthalpy, Helmholtz free energy, volume, and internal energy. By substituting the Hawking temperature (15) and the corrected Bekenstein entropy (46) into Equations (17) and (18), we obtain the corrected enthalpy and the corrected Helmholtz free energy as follows:

(48) $$\begin{array}{c} H_{c1}=-\frac{-\frac{\pi q^{2}}{r_{+}}+2r_{+}\Lambda -\frac{q^{2}}{3r_{+}^{3}\Omega^{2}}-\frac{1}{3}\pi r_{+}\left(3+2\Lambda r_{+}^{2}\right)\Omega^{2}}{2\pi }, \end{array}$$

(49) $$\begin{array}{c} \begin{array}{cc} F_{c1}= & \frac{1}{24\pi r_{+}^{3}\Omega^{2}}[4q^{2}-4\pi r_{+}^{6}\Lambda \Omega^{4}+18\pi q^{2}r_{+}^{2}\Omega^{2}-6r_{+}^{4}\Omega^{2}\left(4\Lambda -\pi \Omega^{2}\right)-\\ & 3\left(q^{2}-r_{+}^{2}\left(1+2\Lambda r_{+}^{2}\right)\Omega^{2}\right)\ln\left(\frac{q^{2}+r_{+}^{2}\left(1+2\Lambda r_{+}^{2}\right)\Omega^{4}}{16\pi r_{+}^{4}\Omega^{2}}\right)]. \end{array} \end{array}$$

**Figure 2:**
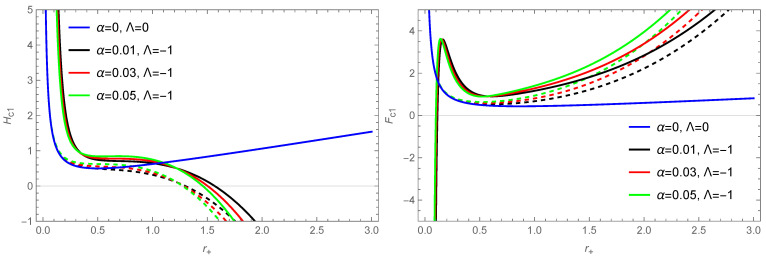
The variation of enthalpy and Helmholtz free energy with the BH’s horizon radius $r_{+}$ for the solution of a BH (10) in the f(R) theory. The dashed line represents the thermodynamic quantities without correction terms, and the solid line represents the corrected thermodynamic quantities. The parameter is taken as q=0.5. The blue curve represents the results for the RN BHs in GR.

Using Equations (17), (18), (48) and (49), we plot the variation of enthalpy and Helmholtz free energy with the horizon radius for the BH in this model in Figure 2. From Figure 2 (left), we find that when the BH’s radius is larger, the corrected enthalpy decreases, which corresponds to an exothermic reaction. According to Figure 2 (right), it can be seen that as the value of parameter α decreases, the value of $F_{c1}$ decreases, which means that the system under consideration changes towards equilibrium. Thus, we cannot extract more work from it.

Combining Equations (19) and (48), we obtain the corrected volume as

(50) $$\begin{array}{c} V_{c1}=8r_{+}\left(1+8\pi \alpha \right)-\frac{64q^{2}\alpha }{3r_{+}^{3}{\left(1+128P\pi \alpha \right)}^{2}}-\frac{8}{3}\pi r_{+}^{3}\left(1+256P\pi \alpha \right). \end{array}$$

Internal energy, as a state function, has a profound significance in thermodynamics. It represents the total energy of the basic structure of a thermodynamic system, which is the sum of the kinetic energy generated by the motion of the particles and the potential energy generated due to the specific structure of these particles [81]. Combining the corrected enthalpy (48) and the corrected volume (50), we can obtain the corrected internal energy as

(51) $$\begin{array}{c} \begin{array}{cc} U_{c1}= & \frac{1}{6}r_{+}\left(16P\left(-3+\pi \left(r_{+}^{2}-24\alpha \right)\right)+4096P^{2}\pi^{2}r_{+}^{2}\alpha -\frac{6\Lambda }{\pi }+\left(3+2r_{+}^{2}\Lambda \right)\Omega^{2}\right)\\ & +\frac{q^{2}}{6r_{+}^{3}}\left(3r_{+}^{2}+\frac{128P\alpha }{{\left(1+128P\pi \alpha \right)}^{2}}+\frac{1}{\pi -16\pi \alpha \Lambda }\right). \end{array} \end{array}$$

**Figure 3:**
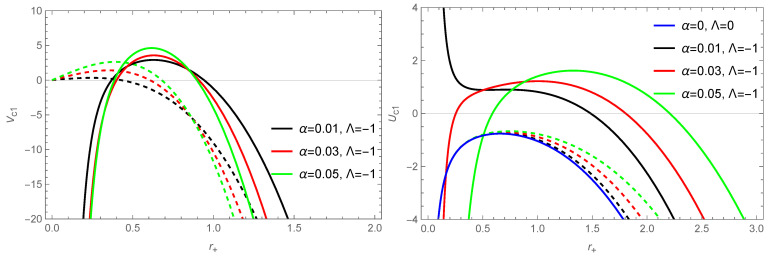
The variation of volume and internal energy with the BH’s horizon radius $r_{+}$ for the solution of a BH (10) in the f(R) theory. The dashed line represents the thermodynamic quantities without correction terms, and the solid line represents the corrected thermodynamic quantities. The parameter is taken as q=0.5. The blue curve represents the result for the RN BH in GR.

According to Equations (21), (22), (50) and (51), the variation of volume and internal energy with $r_{+}$ are shown in Figure 3. From Figure 3 (right), it can be observed that the uncorrected internal energy is negative, whereas the corrected internal energy shows ranges of positive values, indicating that BHs absorb heat from the surrounding environment. Based on the thermodynamic quantities plotted in Figure 3, it is easy to see that for the corrected volume and internal energy, the correction effect caused by thermal fluctuations is more significant for case of the small horizon radius. Considering that the volume needs to be non-negative, it is shown in Figure 3 that this further gives more stringent constraint for the type of black hole, e.g., the requirement on the size of EH’s radius. For our chosen parameter values, α=0.01,q=0.5, and Λ=−1, a non-negative volume corresponds to $0.256<r_{+}<0.949$.

Based on thermodynamic stability, we can know how a system in thermodynamic equilibrium responds to fluctuations in energy, temperature, and other thermodynamic quantities. Stability can be categorized into global stability and local stability. In global stability, we allow the system, which is in equilibrium with a thermodynamic reservoir, to exchange energy with the reservoir. The minimum stage of Gibbs free energy represents the preferred stage of the system [97,106,107,108,109]. By substituting the corrected Helmholtz free energy (49) and the corrected volume (50) into Equation (23), we obtain the corrected Gibbs free energy as follows:

(52) $$\begin{array}{c} \begin{array}{cc} G_{c1}\;=\; & P(8r_{+}\left(1+8\pi \alpha \right)-\frac{64q^{2}\alpha }{3r_{+}^{3}\left(1+128P\pi \alpha \right)}-\frac{8}{3}\pi r_{+}^{3}\left(1+256P\pi \alpha \right))+\frac{1}{24\pi r_{+}^{3}\Omega^{2}}[(4q^{2}-4\pi r_{+}^{6}\Lambda \Omega^{4}+\\ & 18\pi q^{2}r_{+}^{2}\Omega^{2}-6r_{+}^{4}\Omega^{2}\left(4\Lambda -\pi \Omega^{2}\right)-3\left(q^{2}-r_{+}^{2}\left(1+2r_{+}^{2}\Lambda \right)\Omega^{2}\right)\ln(\frac{{\left(q^{2}-r_{+}^{2}\left(1+2r_{+}^{2}\Lambda \right)\Omega^{2}\right)}^{2}}{16\pi r_{+}^{4}\Omega^{2}})]. \end{array} \end{array}$$

**Figure 4:**
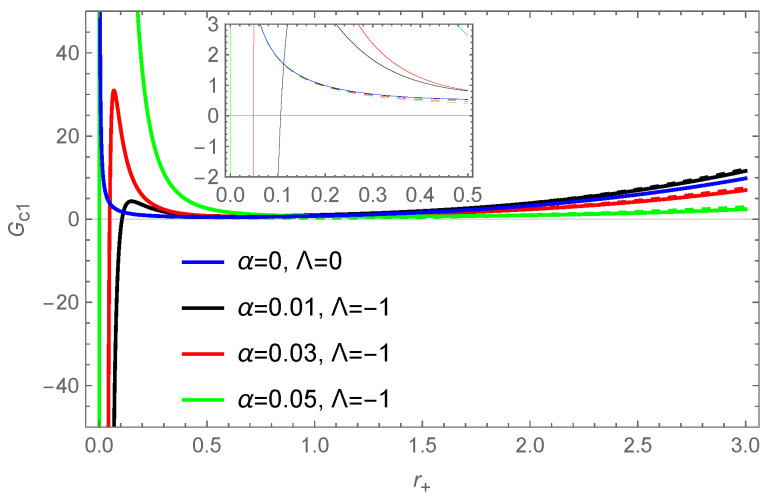
The variation of the Gibbs free energy with the BH’s horizon radius $r_{+}$ for the solution of a BH (10) in the f(R) theory. The dashed line represents the Gibbs free energy without correction terms, and the solid line represents the corrected Gibbs free energy. The parameter is taken as q=0.5. The blue curve represents the result for the RN BH in GR.

Based on the above equation, we plot the variation of the Gibbs free energy of the BH’s horizon radius in Figure 4. Positive Gibbs free energy indicates that non-spontaneous reactions are occurring inside the BH. This means that the system requires more energy to reach an equilibrium position [110]. In Figure 4, it can be observed that the uncorrected Gibbs free energy is always positive, while the corrected Gibbs free energy can be negative for the small BH.

In the canonical ensemble, the local stability of a thermodynamic system can be studied through specific heat. Specific heat contains critical information about the thermal structure of a BH. Its sign determines whether the system is thermally stable or not; in other words, positive values correspond to thermal stability, while negative values indicate instability. By substituting the Hawking temperature (15) and the corrected entropy (50) into Equation (16), we obtain the corrected specific heat as

(53) $$\begin{array}{c} C_{c1}=\frac{2\left(q^{2}\left(-1-\pi r_{+}{‌}^{2}\Omega^{2}\right)-r_{+}{‌}^{4}\Omega^{2}\left(2\Lambda -\pi \left(1+2r_{+}{‌}^{2}\Lambda \right)\Omega^{2}\right)\right)}{3q^{2}+r_{+}{‌}^{2}\left(-1+2r_{+}{‌}^{2}\Lambda \right)\Omega^{2}}. \end{array}$$

**Figure 5:**
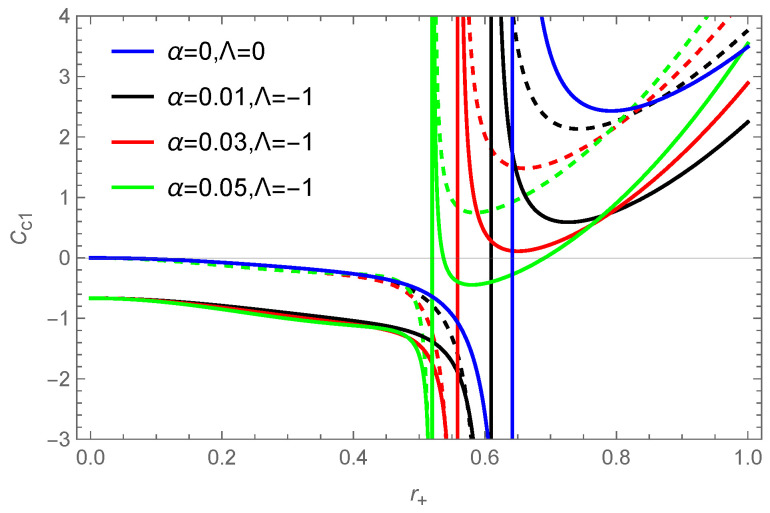
The variation of the specific heat of the BH’s solution (10) in the f(R) theory with the horizon radius $r_{+}$. The dashed line represents the specific heat without the correction term, and the solid line represents the corrected specific heat. The parameter is taken as q=0.5. The blue curve represents the result for the RN BH in GR.

Based on Equation (53), we plot the variation of the specific heat of a BH with the horizon radius $r_{+}$ in Figure 5. In the case of considering thermal fluctuations, it can be observed that for small $r_{+}$, the specific heat of a BH is negative, while for large $r_{+}$, the specific heat is positive, indicating that large BHs are locally thermodynamical stable. It is evident from the figure that both the uncorrected and corrected specific heat show local divergence, indicating that BHs undergo second-order phase transitions [111]. We can further give the range of local stability for the corrected thermodynamics, e.g., for α=0.01, with the BH being locally stable when $r_{+}>0.691$.

#### 3.2.2. $\mathrm{Model}\;\mathrm{II}:\;f\left(R\right)=R+2\gamma \sqrt{R+8\Lambda }$

Following the method in the previous subsection, we can calculate the relevant corrected thermodynamic quantities for Model II as follows:

(54) $$\begin{array}{c} \begin{array}{cc} H_{c2}= & \frac{1}{48\pi r_{+}{‌}^{3}\gamma }[q^{2}\gamma \left(8+24\pi r_{+}{‌}^{2}-3r_{+}\gamma +6r_{+}{‌}^{2}\gamma^{2}\right)\\ & +r_{+}{‌}^{4}\gamma \left(-60\Lambda +\pi \left(12+9r_{+}\gamma +16r_{+}{‌}^{2}\Lambda +18r_{+}{‌}^{3}\gamma \Lambda \right)\right)\\ & +3r_{+}{‌}^{3}\gamma^{2}\left(-1-12\pi q^{2}+2q^{2}\gamma^{2}\right)\ln\left(r\right)+3r_{+}{‌}^{3}\left(\gamma^{2}-2q^{2}\gamma^{4}+4\Lambda \right)\ln\left(1+r_{+}\gamma \right)], \end{array} \end{array}$$

(55) $$\begin{array}{c} \begin{array}{cc} F_{c2}= & -\frac{1}{16\pi }[-2\pi r-\pi r_{+}^{2}\gamma +20r_{+}\Lambda +\frac{8}{3}\pi r_{+}^{3}\Lambda +2\pi r_{+}^{4}\gamma \Lambda +\frac{\ln(64\pi )}{r_{+}}+4r_{+}\Lambda \ln\left(64\pi \right)\\ & -\frac{q^{2}\left(8+36\pi r_{+}^{2}-3r_{+}\gamma +6r_{+}{‌}^{2}\gamma^{2}+6\ln\left(64\pi \right)\right)}{3r_{+}^{3}}+\gamma \left(1+12\pi q^{2}-2q^{2}\gamma^{2}\right)\ln\left(r_{+}\right)\\ & +\frac{\left(-\gamma^{2}+2q^{2}\gamma^{4}-4\Lambda \right)\ln\left(1+r_{+}\gamma \right)}{\gamma }-\frac{\left(-2q^{2}+r_{+}^{2}+4r_{+}^{4}\Lambda \right)\ln(\frac{\left(1+r_{+}\gamma \right){\left(-2q^{2}+r_{+}^{2}+4r_{+}^{4}\Lambda \right)}^{2}}{r_{+}^{2}})}{r_{+}^{3}}], \end{array} \end{array}$$

(56) $$\begin{array}{c} V_{c2}=10r_{+}-\frac{8\pi r_{+}{‌}^{3}}{3}-3\pi r_{+}{‌}^{4}\gamma -\frac{2\ln\left(1+r_{+}\gamma \right)}{\gamma }, \end{array}$$

(57) $$\begin{array}{c} \begin{array}{cc} U_{c2}= & \frac{1}{48\pi r_{+}{‌}^{3}\gamma }[q^{2}\gamma \left(8+24\pi r_{+}^{2}-3r_{+}\gamma +6r_{+}{‌}^{2}\gamma^{2}\right)+3r_{+}{‌}^{4}\beta \left(\pi \left(4+3r_{+}\gamma \right)+12\Lambda \right)+\\ & 3r_{+}\gamma^{2}\left(-1-12\pi q^{2}+2q^{2}\gamma^{2}\right)\ln\left(r\right)-3r_{+}{‌}^{2}\left(-\gamma^{2}+2q^{2}\gamma^{4}+4\Lambda \right)\ln\left(1+r_{+}\gamma \right)], \end{array} \end{array}$$

(58) $$\begin{array}{c} \begin{array}{cc} G_{c2}= & \frac{1}{48\pi r_{+}{‌}^{3}\gamma }[3r_{+}{‌}^{3}\gamma^{2}\left(-1-12\pi q^{2}+2q^{2}\gamma^{2}\right)\ln\left(r_{+}\right)+3r_{+}{‌}^{3}\left(\gamma^{2}-2q^{2}\gamma^{4}+8\Lambda \right)\ln\left(1+r_{+}\gamma \right)\\ & +\gamma \left(6\pi r_{+}{‌}^{4}+3\pi r_{+}^{5}\gamma -120r_{+}{‌}^{4}\Lambda +8\pi r_{+}{‌}^{6}\Lambda +12\pi r_{+}{‌}^{7}\gamma \Lambda -3r_{+}{‌}^{2}\ln\left(64\pi \right)-12r_{+}{‌}^{4}\Lambda \ln\left(64\pi \right)\right)\\ & +3\left(-2q^{2}+r_{+}{‌}^{2}+4r_{+}{‌}^{4}\Lambda \right)\ln(\frac{\left(1+r_{+}\gamma \right){\left(-2q^{2}+r_{+}{‌}^{2}+4r_{+}{‌}^{4}\Lambda \right)}^{2}}{r_{+}{‌}^{2}})], \end{array} \end{array}$$

(59) $$\begin{array}{c} C_{c2}=\frac{\pi r_{+}{‌}^{2}\left(2+3r_{+}\gamma \right)\left(-2q^{2}+r_{+}{‌}^{2}+4r_{+}{‌}^{4}\Lambda \right)}{6q^{2}-r_{+}{‌}^{2}+4r_{+}{‌}^{4}\Lambda }. \end{array}$$

**Figure 6:**
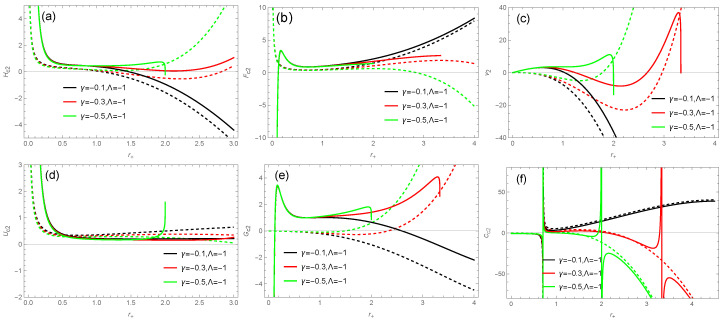
The thermodynamic quantities of the BH’s solution (26) in the f(R) theory as a function of the horizon radius $r_{+}$: (**a**) enthalpy; (**b**) Helmholtz free energy; (**c**) volume; (**d**) internal energy; (**e**) Gibbs free energy; (**f**) specific heat. The dashed lines represent the thermodynamic quantities without the correction term, while the solid lines represent the corrected thermodynamic quantities. The parameter value is taken as q=0.5.

According to Equations (30)–(35) and (54)–(59), the thermodynamic quantities of a BH under $f\left(R\right)=R+2\gamma \sqrt{R+8\Lambda }$ are plotted as a function of the event horizon radius in Figure 6. We observe that the correction term has a smaller effect on internal energy and enthalpy, but a larger effect on free energy, with the impact of the correction term being significant when the event horizon radius is small. According to Figure 6f, we find that the corrected specific heat exhibits local divergence, representing the occurrence of a Hawking–Page phase transition [112]. This second-order phase transition defines the regions of local stability and instability for the BH. From Figure 6c, we can obtain non-negative volume for the corrected quantity when the size of the BH is small, e.g., $r_{+}<1.035$ for γ=−0.1 and $r_{+}<1.999$ for γ=−0.5 in this f(R) model, which is different from the uncorrected result.

## 4. Conclusions

This paper considers two classes of BHs within the framework of the f(R)-modified gravity theory, including the electrodynamic field and the cosmological constant, with a focus on their corrected thermodynamics. Starting from the Hawking temperature and entropy, for two f(R) models, we derived the enthalpy, Helmholtz free energy, volume, internal energy, Gibbs free energy, and specific heat of the BH systems at equilibrium.

The logarithmic entropy correction applicable to small black holes can provide an explanation for the microscopic origin of entropy, which could also help us to solve quantum fluctuation problems related to BH thermodynamic research. Next, considering the presence of small thermal fluctuations around the equilibrium state, we corrected the BH entropy in the framework of the f(R) theory. To study the effect of this correction on entropy, we plotted the entropy of BHs as a function of the event horizon radius for both models. It was found that for the two types of f(R) models we considered, corrected entropy is meaningful for the cases of a negative cosmological constant (anti-de Sitter–RN spacetime) with Λ=−1. Moreover, it was shown that when the BHs’ horizon radii are small, thermal fluctuations have a more significant effect on corrected entropy.

Based on the Hawking temperature and corrected entropy, this paper derived various corrected thermodynamic quantities (e.g., Helmholtz free energy, internal energy, Gibbs free energy, and specific heat) of BHs in the f(R) gravity theory to study the effects of thermal fluctuations. The results indicate that the corrections to Helmholtz free energy and Gibbs free energy caused by thermal fluctuations significantly affect small black holes. In addition, we explored the stability of BHs using specific heat. This study reveals that the corrected BH thermodynamics exhibit local stability for both models and that corrected systems undergo a Hawking–Page phase transition. Considering the requirement concerning the non-negative volume of BHs, we also investigated the constraint on the EH’s radius of BHs.

## Data Availability

No new data were created or analyzed in this study. Data sharing is not applicable to this article.
